# A multiple-time-scale comparative study for the added value of magnetic resonance imaging-based radiomics in predicting pathological complete response after neoadjuvant chemoradiotherapy in locally advanced rectal cancer

**DOI:** 10.3389/fonc.2023.1234619

**Published:** 2023-08-16

**Authors:** Wenjing Peng, Lijuan Wan, Sicong Wang, Shuangmei Zou, Xinming Zhao, Hongmei Zhang

**Affiliations:** ^1^ Department of Radiology, National Cancer Center/National Clinical Research Center for Cancer/Cancer Hospital, Chinese Academy of Medical Sciences and Peking Union Medical College, Beijing, China; ^2^ Department of Pharmaceutical Diagnosis, GE Healthcare, Life Sciences, Beijing, China; ^3^ Department of Pathology, National Cancer Center/National Clinical Research Center for Cancer/Cancer Hospital, Chinese Academy of Medical Sciences and Peking Union Medical College, Beijing, China

**Keywords:** rectal neoplasms, pathological complete response, magnetic resonance imaging, radiomics, neoadjuvant chemoradiotherapy

## Abstract

**Objective:**

Radiomics based on magnetic resonance imaging (MRI) shows potential for prediction of therapeutic effect to neoadjuvant chemoradiotherapy (nCRT) in locally advanced rectal cancer (LARC); however, thorough comparison between radiomics and traditional models is deficient. We aimed to construct multiple-time-scale (pretreatment, posttreatment, and combined) radiomic models to predict pathological complete response (pCR) and compare their utility to those of traditional clinical models.

**Methods:**

In this research, 165 LARC patients undergoing nCRT followed by surgery were enrolled retrospectively, which were divided into training and testing sets in the ratio of 7:3. Morphological features on pre- and posttreatment MRI, coupled with clinical data, were evaluated by univariable and multivariable logistic regression analysis for constructing clinical models. Radiomic parameters were derived from pre- and posttreatment T2- and diffusion-weighted images to develop the radiomic signatures. The clinical-radiomics models were then generated. All the models were developed in the training set and then tested in the testing set, the performance of which was assessed using the area under the receiver operating characteristic curve (AUC). Radiomic models were compared with the clinical models with the DeLong test.

**Results:**

One hundred and sixty-five patients (median age, 55 years; age interquartile range, 47–62 years; 116 males) were enrolled in the study. The pretreatment maximum tumor length, posttreatment maximum tumor length, and magnetic resonance tumor regression grade were selected as independent predictors for pCR in the clinical models. In the testing set, the pre- and posttreatment and combined clinical models generated AUCs of 0.625, 0.842, and 0.842 for predicting pCR, respectively. The MRI-based radiomic models performed reasonably well in predicting pCR, but neither the pure radiomic signatures (AUCs, 0.734, 0.817, and 0.801 for the pre- and posttreatment and combined radiomic signatures, respectively) nor the clinical-radiomics models (AUCs, 0.734, 0.860, and 0.801 for the pre- and posttreatment and combined clinical-radiomics models, respectively) showed significant added value compared with the clinical models (all *P* > 0.05).

**Conclusion:**

The MRI-based radiomic models exhibited no definite added value compared with the clinical models for predicting pCR in LARC. Radiomic models can serve as ancillary tools for tailoring adequate treatment strategies.

## Introduction

Neoadjuvant chemoradiotherapy (nCRT) coupled afterward with surgical resection has been standardly applied in locally advanced rectal cancer (LARC). For LARCs treated by nCRT, approximately 15–27% can obtain pathological complete response (pCR) (1). Concerned with the high operation-related morbidity (surgical complications and bowel and urogenital system dysfunction) and profound lifestyle alteration subsequent to surgery (2, 3), investigators have proposed less invasive or alternative procedures, like a “watch-and-wait” regime or local resection (4, 5) for patients with good response to nCRT. To implement these less invasive approaches safely and efficaciously, precise stratification of patients with pCR is a crucial step.

Magnetic resonance imaging (MRI) has been generally recognized as the standard imaging procedure in the primary evaluation and re-staging of rectal cancer (6, 7). Several MRI characteristics, including tumor volume, signal intensity, and magnetic resonance tumor regression grade (mrTRG) (8–10), have been investigated to predict pCR. However, no consensus exists for any reliable and reproducible methods for accurate prediction before operation. The mrTRG, proposed by the MERCURY research team (11), was demonstrated in several studies (10, 12, 13) to have a significant association with pCR, while a recent meta-analysis reported that mrTRG exhibited superior specificity (93.5%) for pCR, but inferior sensitivity (32.3%) (14).

Radiomics, which provides non-visual information in relation to tumor heterogeneity by extracting many quantitative parameters from digital imaging, has recently been applied to predict treatment response in rectal cancer. A few studies have shown potential results for predicting pCR in LARC using MRI-based texture or radiomic parameters, but substantial limitations have emerged, including the use of single-timepoint models (15–17), single-sequence radiomics analysis (16), and a lack of independent validation (18). Thus, multiple-timepoint models based on multiparametric MRI are required to generalize the definite value of MRI-based radiomics for pCR assessment, in order to promote radiomics into a more practical perspective.

Therefore, our study aimed to develop and validate radiomic models based on multiple MRI timepoints (T2- and diffusion-weighted imaging, T2WI and DWI), and to compare the value of radiomic models in predicting pCR in LARC with traditional clinical models.

## Materials and methods

### Patients

Our research received approval from the institutional ethics committee, accompanied by a waiver for patients’ informed consent due to the retrospective nature of this study. We reviewed consecutive patients who underwent rectal MRI scanning from January 2015 to August 2018 and were diagnosed with rectal cancer by pathology at our institute. The patients were included under the following criteria: (1) rectal adenocarcinoma diagnosed by biopsy; (2) middle or lower rectum located, stage II–III (cT3–4N0M0 or cTxN1–2M0) determined by pretreatment MRI; (3) rectal MRI examinations within two weeks prior to commencing nCRT and at an interval of 4–6 weeks subsequent to nCRT; and (4) completely received nCRT followed by surgical resection. In all, 207 patients were enrolled per the inclusive criteria. The exclusion criteria were as follows: (1) other concomitant tumors (n = 4); (2) mucinous adenocarcinoma (over 50% area of the tumor with high signal on pretreatment T2WI (n = 1); (3) over 8 weeks between the completion of nCRT and the operation (n = 31); and (4) insufficient MRI quality (n = 6). Of those screened, 165 patients were included and allocated to a training and testing cohort in a 7:3 ratio randomly.

Epidemiological parameters and levels of tumor markers were derived from the electronic medical database at our institute, including age, sex, pre- and posttreatment carbohydrate antigen 19-9 (CA19-9), and carcinoembryonic antigen (CEA) levels.

### Neoadjuvant chemoradiotherapy and surgery

All patients received long- or short-course nCRT before surgery. Long-course nCRT was administered as radiotherapy of 45–50.4 Gy to the whole pelvis (5 times per week for 5 weeks) and synchronous chemotherapy (825 mg/m^2^ capecitabine orally, twice a day). Short-course nCRT was administered as radiotherapy of 25 Gy in total, with a fraction of 5 Gy and four cycles of chemotherapy after 7–14 days from completion of radiotherapy (130 mg/m^2^ oxaliplatin intravenously, once a day, on day 1, as well as 1000 mg/m^2^ oral capecitabine, twice a day, during day 1–14). All nCRT was followed by surgical resection, including abdominal-perineal resection, low anterior resection, and Hartmann’s operation.

### Histopathological assessment

Each surgery specimen was evaluated by a pathologist with 21 years’ diagnostic experience in gastrointestinal pathology, abiding by the 8th edition of the American Joint Committee on Cancer’s (AJCC) TNM staging system (19), blinded to imaging data. No residual tumors found in the primary tumor bed and lymph nodes were defined as pCR (ypT0N0).

### MRI parameters and imaging acquisition

MRI scanning was conducted using Discovery MR 750 (GE Healthcare, Chicago, IL), a 3.0-T MRI system with a phased-array surface coil. Raceanisodamine hydrochloride (10 mg) was intramuscularly injected in patients before scanning to suppress bowel motility (except for those with contraindications). Additionally, ultrasound transmission gel (50–60 mL) was injected into the enteric cavity through rectal intubation to highlight the tumor boundary and enhance contrast. Axial T1-weighted imaging (T1WI); axial fat-saturated T2WI (T2WI/FS); axial DWI; and oblique axial, coronal, and sagittal T2WI of two timepoints (pre- and posttreatment) were obtained. The detailed parameters regarding the MRI sequences are presented in **Supplementary Table S1**.

### MRI morphological evaluation

MRI morphological evaluation, including the parameters of distance from tumor to anal verge (DTA), maximum tumor length (MTL), maximum tumor thickness (MTT), circumferential percentage (CP), mrT (ymrT), mrN (ymrN), mesorectal fascia (MRF), extramural vascular invasion (EMVI), and mrTRG was conducted on pre- and posttreatment MR images. These were evaluated by a radiologist with 21 years’ diagnostic experience in gastrointestinal imaging, who was only aware of the pathological results proven by biopsy.

DTA was measured on sagittal T2WI from the anal verge to the tumor’s lowest margin. MTL was recorded as the maximum longitudinal extent from the tumor’s upper to lower margins on sagittal T2WI. MTT and CP were assessed on oblique axial T2WI with maximum tumor dimension. MTT was recorded by the perpendicular distance between the tumor extension’s outer margin and the rectal wall and CP, the tumor invasion’s proportion around the rectal wall with four degrees (degree 1, 0–0.25; 2, > 0.25–0.5; 3, > 0.5–0.75, and 4, > 0.75–1).

The mrT (ymrT) and mrN (ymrN) staging originated from the 8th edition of the AJCC staging system (19). Metastatic lymph node in the primary evaluation and re-staging after nCRT was determined according to the consensus recommended by the European Society of Gastrointestinal and Abdominal Radiology (ESGAR) (7). MRF invasion was defined as the distance equal to or smaller than 1 mm from tumor spiculae to MRF (7). EMVI evaluation was conducted based on a five-point scoring system (20). mrTRGs were assigned in accordance with the description by the MERCURY study group (11): mrTRG1, complete regression, the primary tumor bed without residual tumor signal; mrTRG2, dense low signal fibrosis with minimal tumor signal; mrTRG3, substantial tumor signal; mrTRG4, small areas of fibrosis outgrown by residual tumor; and mrTRG5, extensive residual cancer with no regression or tumor growth. The mrTRG1 was considered pCR, whereas mrTRGs2–5 were considered non-pCR.

### Imaging segmentation and radiomic feature extraction

**Figure 1:**
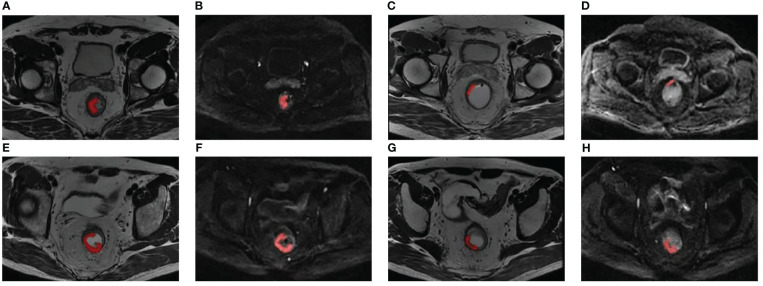
Two examples of segmentation of ROIs on the pre- and posttreatment images. Panels **(A-D)** show the segmentation of a 51-year-old patient with low-rectum adenocarcinoma at a stage of cT3N1M0. **(A, B)** Pretreatment oblique axial T2WI and axial DWI (b = 1000 s/mm^2^); **(C, D)** Posttreatment oblique axial T2WI and axial DWI (b = 1000 s/mm^2^); this patient was demonstrated pCR by surgical pathology. Panels **(E-H)** show the segmentation of a 60-year-old patient with low-rectum adenocarcinoma at a stage of cT3N1M0. **(E, F)** Pretreatment oblique axial T2WI and axial DWI (b = 1000 s/mm^2^); **(G, H)** Posttreatment oblique axial T2WI and axial DWI (b = 1000 s/mm^2^); this patient was demonstrated non-pCR by surgical pathology. DWI, diffusion-weighted imaging; pCR, pathological complete response; ROI, region of interest; T2WI, T2-weighted imaging.

The delineation of regions of interest (ROIs) and radiomic feature extraction were performed on the pre- and posttreatment oblique axial T2WI and axial DWI by using Radcloud version 3.1.0, which was based on the “pyradiomics” package within Python version 3.8.1. Reader 1 (a junior radiologist with three years’ diagnostic experience in gastrointestinal imaging) and reader 2 (a senior radiologist with 16 years’ diagnostic experience in gastrointestinal imaging) conducted a review of each imaging set to reach a consensus over the ROIs. Reader 1 first drew manually on each consecutive tumor-containing slice, which showed intermediate T2WI and high DWI signal in contrast with the normal signal of the muscular layer of the adjacent rectal wall. In some patients, tumor signals were not identified on posttreatment images, and these ROIs were positioned at the location of the tumor bed before treatment (21). **Figure 1** shows two examples of segmentation of ROIs on the pre- and posttreatment images. Reader 2 then examined these ROIs. The two readers would discuss to reach a consensus if there was a discrepancy. They were unaware of the pathological results and clinical data. Imaging normalization weighting coupled with resampling for voxel size (1×1×1 mm^3^) was conducted. Radiomic feature extraction was followed by an automatic procedure. The types of features are listed in **Supplementary Table S2**. There were 1,409 parameters extracted from each modality and 5636 parameters in total were extracted for each patient.

### Radiomic feature selection and signature construction

Z-scores were used to normalize the radiomic features, which aimed at averting the influence of different feature magnitudes. Irrelevant or redundant features were eliminated and 30 parameters with high relevance and low redundancy were reserved using maximum relevance minimum redundancy (mRMR) arithmetic. The performance of 10-fold cross-validation in the training set was calculated and the optimal subset of features was identified using the least absolute shrinkage and selection operator (LASSO) by comparing the results. The values of the tuning parameters (λ) were then determined. Each patient’s pre- and posttreatment radscores were calculated using a weighted linear combination of these selected predictors. The pre- and posttreatment radscores, regarded as two independent radiomic signatures, were compared by Mann–Whitney U test between pCR and non-pCR to explore the significance, respectively. The combined radiomic signature was generated by integrating the pre- and posttreatment radscores using multivariable logistic regression (selection method, Backward: LR).

### Statistical analysis

We employed R (version 4.1.1, R Foundation, Vienna, Austria) and IBM SPSS Statistics (version 25.0, Chicago, IL) to conduct the statistical analyses. Clinical characteristics including demographic data, levels of tumor markers, and MRI morphological parameters were analyzed. The Shapiro–Wilk test was performed for normality assessment. The difference in continuous normally distributed variables was analyzed using the independent t-test between pCR and non-pCR groups, whereas continuous non-normally distributed variables were analyzed using the Mann–Whitney U test. Categorical data were evaluated with the χ^2^ test or Fisher’s exact test. Two-sided *P*-values < 0.05 were considered statistically significant.

The clinical variables were assessed by univariable logistic regression analysis of pCR and non-pCR to explore the significance. The significant variables were then analyzed by multivariable logistic regression (selection method, Backward: LR) to identify the independent predictors to construct the pretreatment, posttreatment, and combined clinical models. The clinical-radiomics models were established in the same way, except for adding the pre- and posttreatment radscores as independent radiomic signatures. All models were established based on the training group and validated by the testing group. The utility of models was evaluated using the areas under the receiver operating characteristic curves (AUCs). Bootstrapping was used to generate 95% confidence intervals (CIs). The DeLong test was conducted to compare the AUCs between models.

**Figure 2:**
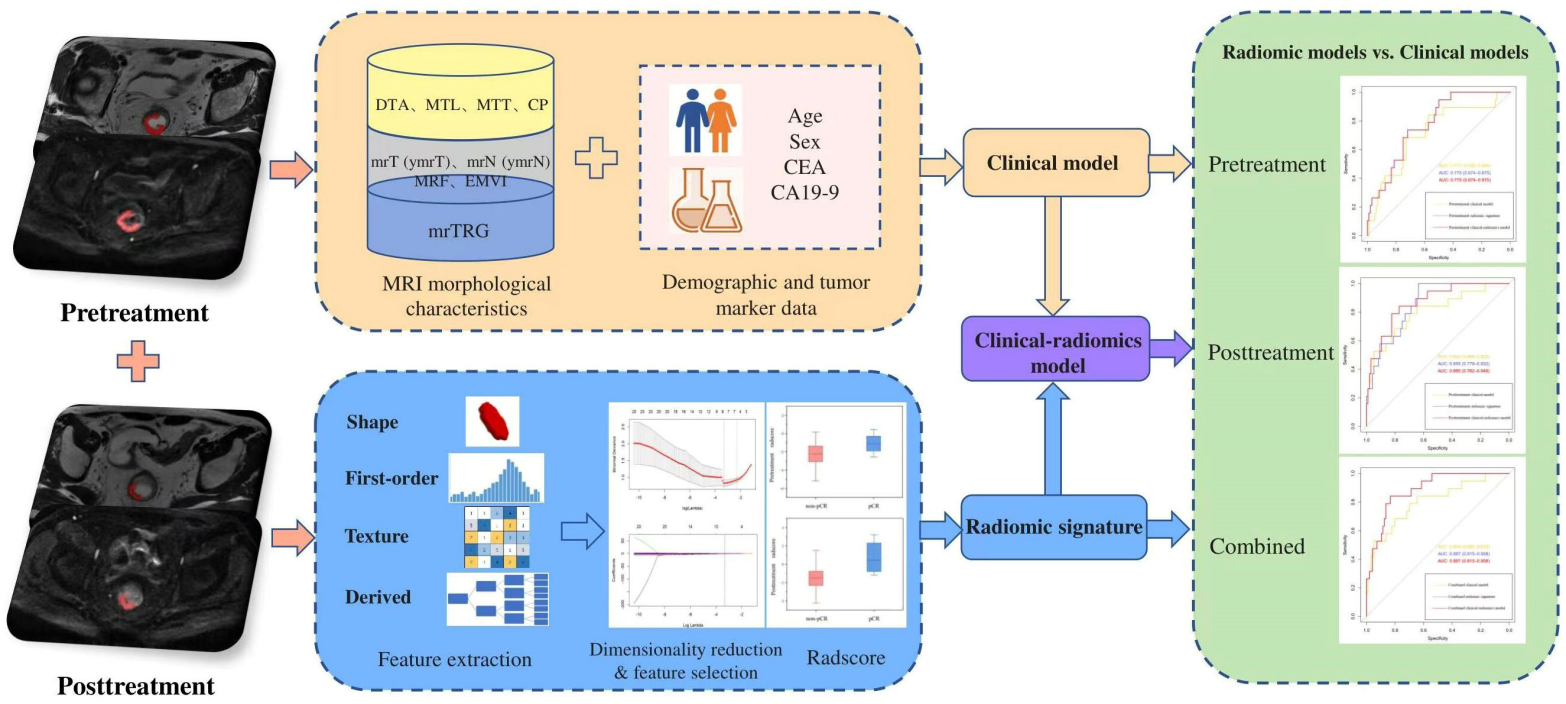
The overall workflow of the comparative study. CA19-9, carbohydrate antigen 19-9; CEA, carcinoembryonic antigen; CP, circumferential percentage; DTA, distance from tumor to anal verge; EMVI, extramural vascular invasion; MRF, mesorectal fascia; MRI, magnetic resonance imaging; mrTRG, magnetic resonance tumor regression grade; MTL, maximum tumor length; MTT, maximum tumor thickness; pCR, pathological complete response.

The overall workflow of the comparative study is presented in **Figure 2**.

## Results

### Patients

One hundred and sixty-five patients (median age, 55 years; age interquartile range, 47–62 years; 116 males) were enrolled in the study. An amount of 115 patients (median age, 56 years; age interquartile range, 50–62 years; 82 males) were allocated to the training cohort, whereas 50 patients (median age, 54 years; age interquartile range, 44–61 years; 34 males) were assigned to the testing cohort. There were no significant differences in clinical variables when comparing the testing and training cohorts (**Tables 1**, **2**). No significant difference was found between the pCR prevalence (16.5% [19/115] vs. 14.0% [7/50], *P* = 0.683) in two cohorts.

**Table 1:** Patients’ clinical characteristics in the training and testing sets.

Characteristic	Training set (n=115)	Testing set (n=50)	*P*
Age (years)	56 (50–62)	54 (44–61)	0.341^a^
Sex			0.669^b^
Male	82 (71%)	34 (68%)	
Female	33 (29%)	16 (32%)	
Pretreatment CEA (ng/mL)			0.409^b^
< 5	61 (53%)	30 (60%)	
≥ 5	54 (47%)	20 (40%)	
Posttreatment CEA (ng/mL)			0.052^b^
< 5	95 (83%)	47 (94%)	
≥ 5	20 (17%)	3 (6%)	
Pretreatment CA19-9 (U/mL)			0.457^b^
< 30	96 (83%)	44 (88%)	
≥ 30	19 (17%)	6 (12%)	
Posttreatment CA19-9 (U/mL)			0.107^c^
< 30	107 (93%)	50 (100%)	
≥ 30	8 (7%)	0 (0%)	
Neoadjuvant chemoradiotherapy			0.511^b^
Long-course chemoradiotherapy	57 (50%)	22 (44%)	
Short-course radiotherapy plus chemotherapy	58 (50%)	28 (56%)	
Surgery			1
Low anterior resection	53 (46%)	23 (46%)	
Abdominal-perineal resection	59 (51%)	26 (52%)	
Hartmann’s operation	3 (3%)	1 (2%)	

Age is expressed as median with interquartile range in parentheses; other measurements are expressed as numbers of patients with percentages in parentheses. CA19-9, carbohydrate antigen 19-9; CEA, carcinoembryonic antigen.

a : Mann–Whitney U test.

b : χ^2^ test.

c : Fisher’s exact test.

**Table 2:** Pre- and posttreatment MRI morphological characteristics of patients in the training and testing sets.

Characteristic	Training set (n=115)	Testing set (n=50)	*P*
Pretreatment DTA, mm	53.4 (35.9–73.6)	44.6 (33.8–67.1)	0.169^a^
Posttreatment DTA, mm	58.4 (41.0–77.3)	49.6 (41.4–69.7)	0.227^a^
Pretreatment MTL, mm	45.4 (37.8–53.3)	43.6 (34.4–58.3)	0.712^a^
Posttreatment MTL, mm	23.5 (16.3–30.6)	24.5 (15.7–30.3)	0.994^a^
Pretreatment MTT, mm	18.2 (15.9–22.2)	19.4 (15.2–24.7)	0.606^a^
Posttreatment MTT, mm	10.4 (7.8–13.2)	10.5 (6.9–12.7)	0.492^a^
Pretreatment CP			0,978^c^
0–0.25	2 (2%)	1 (2%)	
>0.25–0.5	23 (20%)	11 (22%)	
>0.5–0.75	48 (42%)	20 (40%)	
>0.75–1	42 (37%)	18 (36%)	
Posttreatment CP			0,639^b^
0–0.25	31 (27%)	15 (30%)	
>0.25–0.5	58 (50%)	20 (40%)	
>0.5–0.75	17 (15%)	10 (20%)	
>0.75–1	9 (8%)	5 (10%)	
Pretreatment mrT stage			0.094^c^
1	0 (0%)	0 (0%)	
2	1 (1%)	0 (0%)	
3a	15 (13%)	6 (12%)	
3b	70 (61%)	38 (76%)	
3c	22 (19%)	2 (4%)	
3d	4 (3%)	3 (6%)	
4	3 (3%)	1 (2%)	
Posttreatment mrT (ymrT) stage			0.121^c^
0	12 (10%)	6 (12%)	
1	1 (1%)	2 (4%)	
2	16 (14%)	7 (14%)	
3	71 (62%)	34 (68%)	
4	15 (13%)	1 (2%)	
Pretreatment mrN stage			0.684^b^
0	28 (24%)	15 (30%)	
1	51 (44%)	22 (44%)	
2	36 (31%)	13 (26%)	
Posttreatment mrN (ymrN) stage			0.648^c^
0	78 (68%)	38 (76%)	
1	34 (30%)	11 (22%)	
2	3 (3%)	1 (2%)	
Pretreatment MRF			0.571^b^
Positive	34 (30%)	17 (34%)	
Posttreatment MRF			1^c^
Positive	7 (6%)	3 (6%)	
Pretreatment EMVI			0.835^b^
Positive	67 (58%)	30 (60%)	
Posttreatment EMVI			0.786^b^
Positive	18 (16%)	7 (14%)	
mrTRG			0.861^c^
1	24 (21%)	13 (26%)	
2	72 (63%)	28 (56%)	
3	16 (14%)	8 (16%)	
4	3 (3%)	1 (2%)	
5	0 (0%)	0 (0%)	

Pre- and posttreatment DTA, MTL, and MTT are expressed as median with interquartile range in parentheses; other measurements are expressed as numbers of patients with percentages in parentheses. CP, circumferential percentage; DTA, distance from tumor to anal verge; EMVI, extramural vascular invasion; MRF, mesorectal fascia; MRI, magnetic resonance imaging; mrTRG, magnetic resonance tumor regression grade; MTL, maximum tumor length; MTT, maximum tumor thickness.

a : Mann–Whitney U test.

b : χ^2^ test.

c : Fisher’s exact test.

### Radiomic feature selection and signature construction

**Figure 3:**
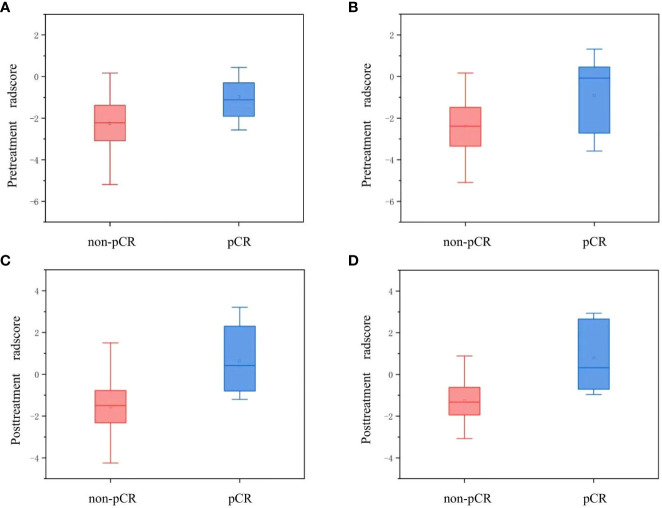
Box plots for the pre- and posttreatment radscores of the pCR and non-pCR groups. Panels **(A, B)** show the pretreatment radscores in the training and testing sets, respectively. Panels **(C, D)** show the posttreatment radscores in the training and testing sets, respectively. pCR, pathological complete response.

A subset of three pretreatment and eight posttreatment radiomic parameters was confirmed separately as the optimal candidate predictor for the radiomic signatures. Detailed information regarding the contributing weight of the selected radiomic features is shown in **Supplementary Figure S1**. Both the pre- and posttreatment radscores of pCR patients in the training set were larger than those of non-pCR patients (median: pretreatment radscore, -1.11 vs. -2.22, *P* = 0.011; posttreatment radscore, 0.42 vs. -1.50, *P* < 0.001). These were verified by the testing cohort (median: pretreatment radscore, -0.07 vs. -2.39, *P* = 0.415; posttreatment radscore, 0.32 vs. -1.32, *P* = 0.014). **Figure 3** shows box plots of the pre- and posttreatment radscores in the training and testing sets for the pCR and non-pCR groups. The pre- and posttreatment radscores were enrolled in the combined radiomic signature as independent predictors. The pretreatment, posttreatment, and combined radiomic signatures exhibited AUCs of 0.775–0.887 for the training group and 0.734–0.817 for the testing group.

### Model development and validation

In the training set, the pretreatment MTL (*P* = 0.011), posttreatment MTL (*P* = 0.001), posttreatment MTT (*P* = 0.025), posttreatment mrT stage (ymrT) (*P* = 0.046), and mrTRG (*P* < 0.001) between the pCR and non-pCR groups were significantly different by the univariable logistic regression analysis. The pretreatment MTL, the only predictor identified in the pretreatment clinical model, achieved the lowest AUCs both in the training set (AUC, 0.717; 95% CI, 0.587–0.848) and the testing set (AUC, 0.625; 95% CI, 0.375–0.874). The posttreatment MTL (odds ratio [OR], 0.912; 95% CI, 0.848–0.980) and mrTRG (OR, 6.064; 95% CI, 1.933–19.020) were selected by the multivariable logistic regression analysis as independent predictors both in the posttreatment and combined clinical models, which achieved the same AUCs of 0.804 (95% CI, 0.685–0.922) and 0.842 (95% CI, 0.709–0.975) separately for the training and testing cohorts.

**Figure 4:**
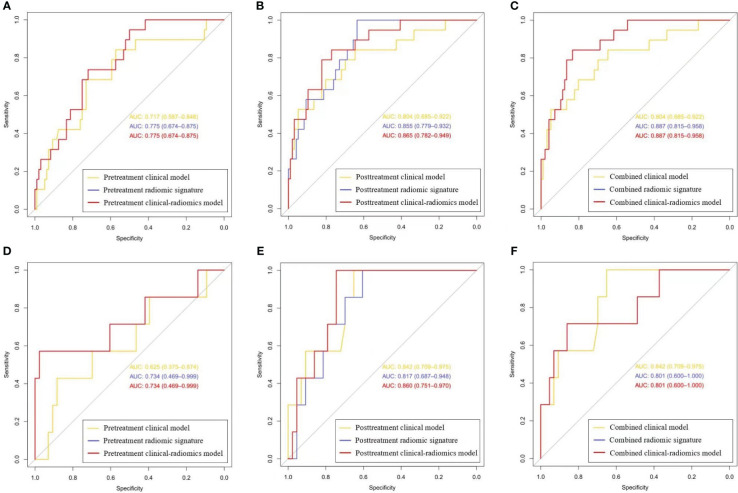
The receiver operating characteristic curves for the clinical, radiomics, and clinical-radiomics models of three timepoints. **(A-C)** Curves for the pretreatment, posttreatment, and combined models in the training set, respectively; **(D-F)** Curves for the pretreatment, posttreatment, and combined models in the testing set, respectively. (Curves for the pretreatment and combined clinical-radiomics models coincide with those of the pretreatment and combined radiomic signatures, respectively, which are displayed in red).

In three clinical-radiomics models, the characteristics enrolled as independent predictors were separate as follows: the pretreatment radscore for the pretreatment clinical-radiomics model; the posttreatment MTL (OR, 0.929; 95% CI, 0.862–1) and posttreatment radscore (OR, 2.236; 95% CI, 1.471–3.400) for the posttreatment clinical-radiomics model; and the pretreatment radscore (OR, 2.370; 95% CI, 1.217–4.615) and posttreatment radscore (OR, 2.153; 95% CI, 1.426–3.251) for the combined clinical-radiomics model. The clinical-radiomics models of three timepoints achieved AUCs of 0.775–0.887 for the training group and 0.734–0.860 for the testing group. **Table 3** and **Figure 4** show the AUCs of the models.

**Table 3:** The areas under the receiver operating characteristic curves of multiple-time-scale models.

AUC	Clinical model	Radiomic signature	Clinical-radiomics model
Training set			
Pretreatment	0.717 (0.587–0.848)	0.775 (0.674–0.875)	0.775 (0.674–0.875)
Posttreatment	0.804 (0.685–0.922)	0.855 (0.779–0.932)	0.865 (0.782–0.949)
Combined	0.804 (0.685–0.922)	0.887 (0.815–0.958)	0.887 (0.815–0.958)
Testing set			
Pretreatment	0.625 (0.375–0.874)	0.734 (0.469–0.999)	0.734 (0.469–0.999)
Posttreatment	0.842 (0.709–0.975)	0.817 (0.687–0.948)	0.860 (0.751–0.970)
Combined	0.842 (0.709–0.975)	0.801 (0.600–1)	0.801 (0.600–1)

Data in parentheses are 95% CIs. AUC, area under the receiver operating characteristic curve; CI, confidence interval.

The combined radiomic signature, as well as the combined clinical-radiomics model, achieved the highest AUC in the training group (AUC, 0.887; 95% CI, 0.815–0.958), while it was the posttreatment clinical-radiomics model (AUC, 0.860; 95% CI, 0.751–0.970) in the testing group. Furthermore, comparisons of MRI-based radiomic models and clinical models showed that neither the pure radiomic signatures nor the clinical-radiomics models of three timepoints were significantly different from the clinical models (all *P* > 0.05), both in the training and testing sets. Also, there were no significant differences when comparing the pure radiomic signatures with clinical-radiomics models of three timepoints (all *P* > 0.05). **Table 4** illustrates the comparison between the models.

**Table 4:** Comparison of the areas under the receiver operating characteristic curves of models on different timepoints.

*P*	Clinical vs. Radiomics	Clinical vs. Clinical-radiomics	Radiomics vs. Clinical-radiomics
Training set			
Pretreatment	0.243	0.243	1
Posttreatment	0.342	0.069	0.709
Combined	0.101	0.101	1
Testing set			
Pretreatment	0.369	0.369	1
Posttreatment	0.627	0.684	0.09
Combined	0.660	0.660	1

## Discussion

The precise stratification of LARC patients with pCR after nCRT has become a crucial issue, because they can consider less invasive procedures, like a “watch-and-wait” regime or local resection. MRI-based radiomics shows potential for predicting pCR, but the thorough comparison between radiomics and traditional models is deficient. In this study, we developed and validated multiple time-scale (pretreatment, posttreatment, and combined) radiomic models based on MRI to predict pCR and compared their utility to those of traditional clinical models. Radiomic models performed reasonably well for predicting pCR in LARC. However, neither the pure radiomic signatures nor the clinical-radiomics models of three timepoints showed a definite added value to the traditional clinical models.

The pretreatment radiomic signature generated moderate AUCs of 0.734–0.775 to predict pCR, which were concordant with the results of previous studies (18, 22, 23). Meanwhile, the posttreatment and combined radiomic signatures obtained higher AUCs (0.801–0.887) than the pretreatment one. The superiority of posttreatment imaging corresponded well with the former investigation (24), which is theoretically directly linked to pathological results. To scrutinize the pure radiomic signatures, consistent with prior studies (25), high weights of high-order radiomic features were included in the models, with 3/3 of the pretreatment and 4/8 of the posttreatment features being the wavelet features, which reflect the change rate of the pixel value in the frequency domain (26), representing the complexity and heterogeneity of tumors and can better predict pCR.

Clinical models performed inferiorly to well in our research. The pretreatment clinical model based on the single MTL got the lowest prediction performance (AUC, 0.625) in the testing set, which indicated the predicting insufficiency of pure pretreatment morphological features. But even so, the pretreatment radiomic models (including both the pure radiomic signature and clinical-radiomics model) didn’t perform significantly superior to the clinical model. The posttreatment clinical model generated higher AUCs than the pretreatment one and the combined clinical model only reserved the posttreatment clinical features as the independent predictors after multivariable logistic regression selection with the method of Backward, which implied the superior predicting utility of posttreatment clinical features. Both the posttreatment and combined clinical models were based on the posttreatment MTL and mrTRG. Posttreatment MTL has been recognized as an effective morphological predictor in assessing pCR as former studies reported (24, 27, 28). Another promising predictor was mrTRG, which reflected the tumor signal status after treatment and highly correlated with the tumor response (10, 29). In the comparison of the posttreatment radiomic models (including both the pure radiomic signature and clinical-radiomics model) with the clinical model, there was still no added significant value that emerged, which was the same as the combined models. Our results were consistent with a handful of previous reports. Shi et al. reported that a pretreatment radiomic model could predict pCR but showed no significant difference from the clinical model. However, the conclusion lacked independent validation (18). Bulens et al. illustrated in an external validation cohort that neither the pure radiomic model based on pre- and posttreatment MRI nor the clinical-radiomics model outperformed the clinical model in predicting (near-)pCR. However, the study did not conduct further stratification research by timepoint (30).

The strength of our study resides in our multiple-timepoint and multiple-modality comparative analysis in the field of radiomics, which is few in the current research. Recent studies employing radiomics to predict pCR in LARC have been an exponential growth, while few of them clarified the usefulness of MRI-based radiomic models, especially compared with the traditional clinical ones. Our study promotes radiomics into a more applicable perspective and gets the conclusions with general applicability and realistic instruction. Since the ambiguous superiority over the clinical models and the laborious and intricate process during radiomic analyzing, the application of radiomic modeling is far from routine in the clinical practice. Clinical parameters, including the emerging ones, such as histopathological, immunohistochemical, and genetic, still deserve further investigation.

It is worth noting that radiomics is not devoid of any advantages. Considering the realistic diagnostic procedures in the clinical practice that mrTRG can vary in doctors with different experience and the final diagnosis are always concluded by the senior one in a two-observer review, we took mrTRG results evaluated by a senior radiologist into the analysis. Consistent with the former literature (specificity, 92–98%; sensitivity, 0–59%) (14), mrTRG in our study obtained moderate to high specificity (0.791–0.865) and low sensitivity (0.571–0.579). In contrast, expert input cannot be a requirement in radiomics analysis. It was reported (31) that significant predictive performance can be achieved regardless of whether radiomics ROI segmentation was done by an experienced radiologist or a junior resident. And our radiomic ROI segmentation was conducted by a junior one. In this case, radiomics can serve as a supplementary tool in senior-absent situations to add confidence in treatment response assessment and help tailor the treatment strategies adequately.

There were several limitations in this research. First, it was a retrospective analysis with a limited scale of datasets in a single institute, which might carry inherent selection bias. Prospective and multicenter external validation deserves further investigation in the future. Second, many factors can potentially affect the reproducibility of radiomic features, such as scanning sequence, data acquisition, image preprocessing, segmentation strategy, and feature extraction tools. In this study, we performed several measures (eg ROIs were delineated by two radiologists in consensus and the whole-volume segmentation) to improve feature reproducibility. Therefore, we did not evaluate the interobserver and intraobserver reproducibility of segmentation. Third, other functional sequences such as dynamic contrast-enhanced MRI (DCE-MRI) and apparent diffusion coefficient (ADC) maps were not enrolled in our study. DCE-MRI is not routinely applied in the rectal MRI examination; ADC maps are vulnerable, with geometric distortion and sensitivity to susceptibility artifacts.

In conclusion, our study showed that MRI-based radiomic models performed reasonably well for the prediction of pCR in LARC, but exhibited no definite added value compared to the traditional clinical models. Radiomic models can serve as ancillary tools for selecting candidate pCR patients and tailoring adequate treatment strategies.

## Data availability statement

The original contributions presented in the study are included in the article/**Supplementary Material**. Further inquiries can be directed to the corresponding author.

## Ethics statement

The studies involving human participants were reviewed and approved by National Cancer Center/National Clinical Research Center for Cancer/Cancer Hospital, Chinese Academy of Medical Sciences and Peking Union Medical College. Written informed consent for participation was not required for this study in accordance with the national legislation and the institutional requirements. Written informed consent was not obtained from the individual(s) for the publication of any potentially identifiable images or data included in this article.

## Author contributions

WP and HZ conceived this study. WP, LW, SZ, and HZ performed the research and collected the data. WP, SW, and HZ analyzed and interpreted the data. WP wrote and edited the manuscript. HZ and XZ revised the manuscript for important intellectual content. All authors contributed to the article and approved the submitted version.

## References

[1] Maas M Nelemans PJ Valentini V Das P Rödel C Kuo LJ. Long-term outcome in patients with a pathological complete response after chemoradiation for rectal cancer: a pooled analysis of individual patient data. Lancet Oncol (2010) 11(9):835–44. doi: 10.1016/S1470-2045(10)70172-8 20692872

[2] Hupkens BJP Martens MH Stoot JH Berbee M Melenhorst J Beets-Tan RG. Quality of life in rectal cancer patients after chemoradiation: watch-and-wait policy versus standard resection - a matched-controlled study. Dis Colon Rectum (2017) 60(10):1032–40. doi: 10.1097/DCR.0000000000000862 28891846

[3] Renehan AG Malcomson L Emsley R Gollins S Maw A Myint AS. Watch-and-wait approach versus surgical resection after chemoradiotherapy for patients with rectal cancer (the OnCoRe project): a propensity-score matched cohort analysis. Lancet Oncol (2016) 17(2):174–83. doi: 10.1016/S1470-2045(15)00467-2 26705854

[4] Habr-Gama A Perez RO. The surgical significance of residual mucosal abnormalities in rectal cancer following neoadjuvant chemoradiotherapy. Br J Surg (2012) 99(11):1601. doi: 10.1002/bjs.8946 22351592

[5] Fernandez LM São Julião GP Figueiredo NL Beets GL van der Valk MJM Bahadoer RR. Conditional recurrence-free survival of clinical complete responders managed by watch and wait after neoadjuvant chemoradiotherapy for rectal cancer in the international watch & wait database: a retrospective, international, multicentre registry study. Lancet Oncol (2021) 22(1):43–50. doi: 10.1016/S1470-2045(20)30557-X 33316218

[6] Horvat N Carlos Tavares Rocha C Clemente Oliveira B Petkovska I Gollub MJ. MRI Of rectal cancer: tumor staging, imaging techniques, and management. RadioGraphics (2019) 39(2):367–87. doi: 10.1148/rg.2019180114 PMC6438362 30768361

[7] Beets-Tan RGH Lambregts DMJ Maas M Bipat S Barbaro B Curvo-Semedo L. Magnetic resonance imaging for clinical management of rectal cancer: updated recommendations from the 2016 European society of gastrointestinal and abdominal radiology (ESGAR) consensus meeting. Eur Radiol (2018) 28(4):1465–75. doi: 10.1007/s00330-017-5026-2 PMC5834554 29043428

[8] Lambregts DM Rao SX Sassen S Martens MH Heijnen LA Buijsen J. MRI And diffusion-weighted MRI volumetry for identification of complete tumor responders after preoperative chemoradiotherapy in patients with rectal cancer: a bi-institutional validation study. Ann Surg (2015) 262(6):1034–9. doi: 10.1097/SLA.0000000000000909 25211270

[9] Wan L Zhang C Zhao Q Meng Y Zou S Yang Y. Developing a prediction model based on MRI for pathological complete response after neoadjuvant chemoradiotherapy in locally advanced rectal cancer. Abdom Radiol (NY) (2019) 44(9):2978–87. doi: 10.1007/s00261-019-02129-6 31327039

[10] Suzuki C Halperin SK Nilsson PJ Martling A Holm T. Initial magnetic resonance imaging tumour regression grade (mrTRG) as response evaluation after neoadjuvant treatment predicts sustained complete response in patients with rectal cancer. Eur J Surg Oncol (2022) 48(7):1643–9. doi: 10.1016/j.ejso.2022.02.012 35272899

[11] Patel UB Taylor F Blomqvist L George C Evans H Tekkis P. Magnetic resonance imaging-detected tumor response for locally advanced rectal cancer predicts survival outcomes: MERCURY experience. J Clin Oncol (2011) 29(28):3753–60. doi: 10.1200/JCO.2011.34.9068 21876084

[12] Fayaz MS Demian GA Fathallah WM Eissa HE El-Sherify MS Abozlouf S. Significance of magnetic resonance imaging-assessed tumor response for locally advanced rectal cancer treated with preoperative long-course chemoradiation. J Glob Oncol (2016) 2(4):216–21. doi: 10.1200/JGO.2015.001479 PMC5497621 28717704

[13] Popita AR Lisencu C Rusu A Popita C Cainap C Irimie A. MRI Evaluation of complete and near-complete response after neoadjuvant therapy in patients with locally advanced rectal cancer. Diagn (Basel) (2022) 12(4):921. doi: 10.3390/diagnostics12040921 PMC9027294 35453969

[14] Jang JK Choi SH Park SH Kim KW Kim HJ Lee JS. MR tumor regression grade for pathological complete response in rectal cancer post neoadjuvant chemoradiotherapy: a systematic review and meta-analysis for accuracy. Eur Radiol (2020) 30(4):2312–23. doi: 10.1007/s00330-019-06565-2 31953656

[15] Cui Y Yang X Shi Z Yang Z Du X Zhao Z. Radiomics analysis of multiparametric MRI for prediction of pathological complete response to neoadjuvant chemoradiotherapy in locally advanced rectal cancer. Eur Radiol (2019) 29(3):1211–20. doi: 10.1007/s00330-018-5683-9 30128616

[16] Petkovska I Tixier F Ortiz EJ Golia Pernicka JS Paroder V Bates DD. Clinical utility of radiomics at baseline rectal MRI to predict complete response of rectal cancer after chemoradiation therapy. Abdom Radiol (NY) (2020) 45(11):3608–17. doi: 10.1007/s00261-020-02502-w PMC7572430 32296896

[17] Shin J Seo N Baek SE Son NH Lim JS Kim NK. MRI Radiomics model predicts pathologic complete response of rectal cancer following chemoradiotherapy. Radiology (2022) 303(2):351–8. doi: 10.1148/radiol.211986 35133200

[18] Shi L Zhang Y Nie K Sun X Niu T Yue N. Machine learning for prediction of chemoradiation therapy response in rectal cancer using pre-treatment and mid-radiation multi-parametric MRI. Magn Reson Imaging (2019) 61:33–40. doi: 10.1016/j.mri.2019.05.003 31059768 PMC7709818

[19] Weiser MR. AJCC 8th edition: colorectal cancer. Ann Surg Oncol (2018) 25(6):1454–5. doi: 10.1245/s10434-018-6462-1 29616422

[20] Zhang XY Wang S Li XT Wang YP Shi YJ Wang L. MRI Of extramural venous invasion in locally advanced rectal cancer: relationship to tumor recurrence and overall survival. Radiology (2018) 289(3):677–85. doi: 10.1148/radiol.2018172889 30152742

[21] Blazic IM Lilic GB Gajic MM. Quantitative assessment of rectal cancer response to neoadjuvant combined chemotherapy and radiation therapy: comparison of three methods of positioning region of interest for ADC measurements at diffusion-weighted MR imaging. Radiology (2017) 282(2):418–28. doi: 10.1148/radiol.2016151908 27253423

[22] Shaish H Aukerman A Vanguri R Spinelli A Armenta P Jambawalikar S. Radiomics of MRI for pretreatment prediction of pathologic complete response, tumor regression grade, and neoadjuvant rectal score in patients with locally advanced rectal cancer undergoing neoadjuvant chemoradiation: an international multicenter study. Eur Radiol (2020) 30(11):6263–73. doi: 10.1007/s00330-020-06968-6 32500192

[23] Song M Li S Wang H Hu K Wang F Teng H. MRI Radiomics independent of clinical baseline characteristics and neoadjuvant treatment modalities predicts response to neoadjuvant therapy in rectal cancer. Br J Cancer (2022) 127(2):249–57. doi: 10.1038/s41416-022-01786-7 PMC9296479 35368044

[24] Liu Z Zhang XY Shi YJ Wang L Zhu HT Tang Z. Radiomics analysis for evaluation of pathological complete response to neoadjuvant chemoradiotherapy in locally advanced rectal cancer. Clin Cancer Res (2017) 23(23):7253–62. doi: 10.1158/1078-0432.CCR-17-1038 28939744

[25] Wan L Peng W Zou S Ye F Geng Y Ouyang H. MRI-Based delta-radiomics are predictive of pathological complete response after neoadjuvant chemoradiotherapy in locally advanced rectal cancer. Acad Radiol (2021) 28:S95–104. doi: 10.1016/j.acra.2020.10.026 33189550

[26] Majeed Alneamy JS Hameed Alnaish Z A Mohd Hashim SZ Hamed Alnaish RA. Utilizing hybrid functional fuzzy wavelet neural networks with a teaching learning-based optimization algorithm for medical disease diagnosis. Comput Biol Med (2019) 112:103348. doi: 10.1016/j.compbiomed.2019.103348 31356992

[27] Pyo DH Choi JY Lee WY Yun SH Kim HC Huh JW. A nomogram for predicting pathological complete response to neoadjuvant chemoradiotherapy using semiquantitative parameters derived from sequential PET/CT in locally advanced rectal cancer. Front Oncol (2021) 11:742728. doi: 10.3389/fonc.2021.742728 34676170 PMC8523984

[28] Wan L Sun Z Peng W Wang S Li J Zhao Q. Selecting candidates for organ-preserving strategies after neoadjuvant chemoradiotherapy for rectal cancer: development and validation of a model integrating MRI radiomics and pathomics. J Magn Reson Imaging (2022) 56(4):1130–42. doi: 10.1002/jmri.28108 35142001

[29] Achilli P Magistro C Abd El Aziz MA Calini G Bertoglio CL Ferrari G. Modest agreement between magnetic resonance and pathological tumor regression after neoadjuvant therapy for rectal cancer in the real world. Int J Cancer (2022) 151(1):120–7. doi: 10.1002/ijc.33975 35191540

[30] Bulens P Couwenberg A Intven M Debucquoy A Vandecaveye V Van Cutsem E. Predicting the tumor response to chemoradiotherapy for rectal cancer: model development and external validation using MRI radiomics. Radiother Oncol (2020) 142:246–52. doi: 10.1016/j.radonc.2019.07.033 PMC6997038 31431368

[31] van Griethuysen JJM Lambregts DMJ Trebeschi S Lahaye MJ Bakers FCH Vliegen RFA. Radiomics performs comparable to morphologic assessment by expert radiologists for prediction of response to neoadjuvant chemoradiotherapy on baseline staging MRI in rectal cancer. Abdom Radiol (NY) (2020) 45(3):632–43. doi: 10.1007/s00261-019-02321-8 31734709

