# Supplementary material for: A multiple-time-scale comparative study for the added value of magnetic resonance imaging-based radiomics in predicting pathological complete response after neoadjuvant chemoradiotherapy in locally advanced rectal cancer
Source: Front Oncol. 2023 Aug 16;13:1234619. doi: 10.3389/fonc.2023.1234619 (PMC10468971; doi:10.3389/fonc.2023.1234619)
Supplement: Supplementary file 1 [file DataSheet_1.docx]

Supplementary Material

**Supplementary Figure S1.** Contributing coefficients of the selected radiomic features in the pretreatment **(A)** and posttreatment **(B)** radiomic signatures.


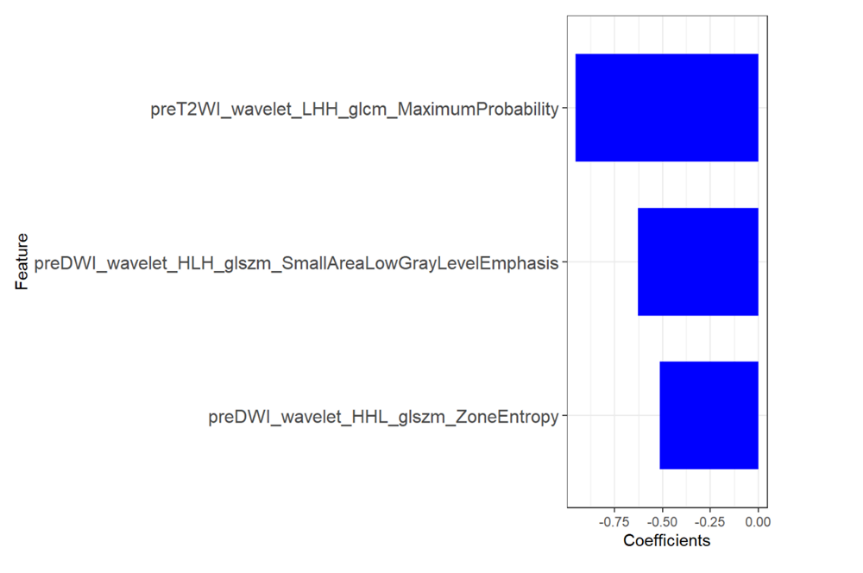
A

B

**
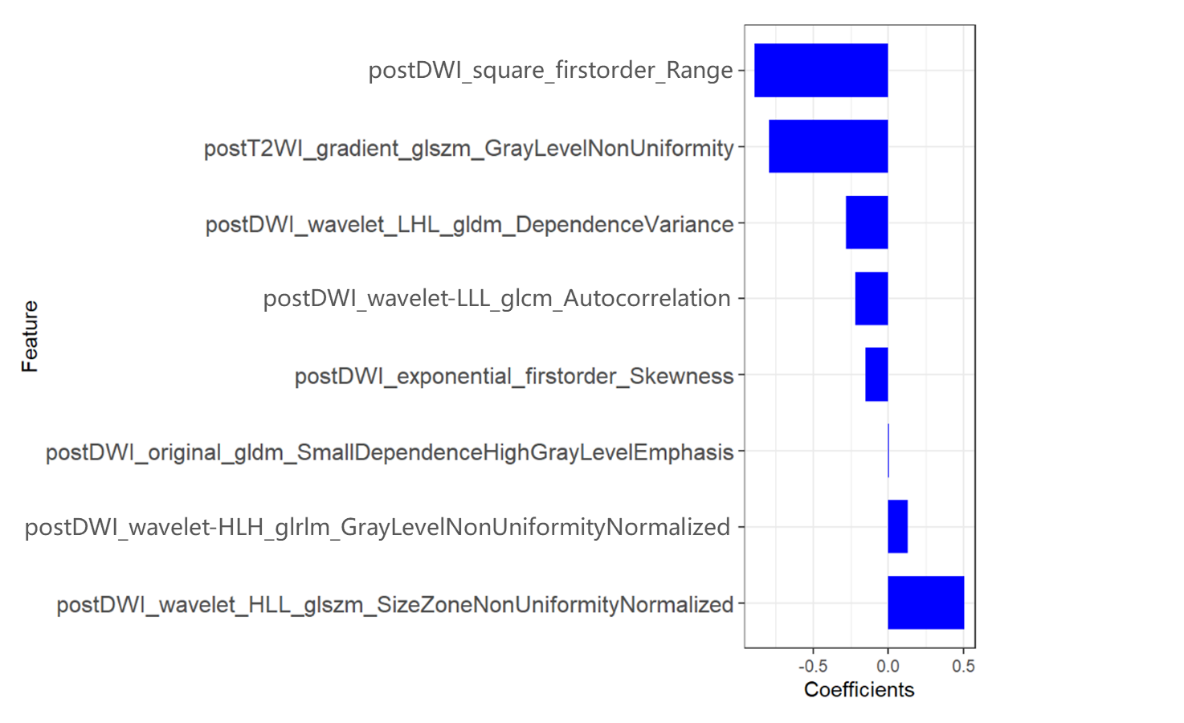
**

### Supplementary Table S1. Parameters for magnetic resonance imaging sequences

| **Parameter** | **Oblique axial T2WI** | **Sagittal T2WI** | **Coronal T2WI** | **Axial T1WI** | **Axial T2WI/FS** | **Axial DWI** |
| --- | --- | --- | --- | --- | --- | --- |
| Repetition time, ms | 4800 | 4800 | 4800 | 560 | 5700 | 2300 |
| Echo time, ms | 115 | 115 | 115 | Min | 85 | Min |
| Field of view, mm | 160 | 240 | 240 | 340 | 340 | 340 |
| Matrix | 320×256 | 320×256 | 320×256 | 288×224 | 288×224 | 160×128 |
| Bandwidth, kHz | 41 | 41 | 41 | 41 | 31 | 250 |
| Echo train length | 21 | 21 | 21 | 4 | 21 | N/A |
| Number of excitations | 4 | 4 | 4 | 2 | 2 | 2 |
| Slice thickness, mm | 3 | 4 | 4 | 5 | 5 | 5 |
| Intersection gap, mm | 0 | 0.4 | 0.4 | 0.5 | 0.5 | 0.5 |
| *b* values, s/mm^2^ | N/A | N/A | N/A | N/A | N/A | 0, 1000 |

DWI, diffusion-weighted imaging; Min, minimum; N/A, not applicable; T1WI, T1-weighted imaging; T2WI, T2-weighted imaging; T2WI/FS, fat-saturated T2-weighted imaging.

**Supplementary Table S2. Types of radiomic features extracted**

| Original features  (n=107) | | | Derived features  (n=1302) | |
| --- | --- | --- | --- | --- |
| Shape features (n = 14) | First-order features (n = 18) | Texture features (n = 75) | | n = (18 + 75) × 14 |
| voxel volume, maximum 3D diameter, mesh volume, major axis length, sphericity, least axis length, elongation, surface volume ratio, maximum 2D diameter (slice), flatness, surface area, minor axis length, maximum 2D diameter (column), maximum 2D diameter (row) | interquartile range, skewness, uniformity, median, energy, robust mean absolute deviation, mean absolute deviation, total energy, maximum, root mean squared, 90 percentile, minimum, entropy, range, variance, 10 percentile, kurtosis, mean | GLCM (n = 24), GLDM (n = 14), GLRLM (n = 16), GLSZM (n = 16), NGTDM (n = 5) | | logarithm, exponential, gradient, square, square root, logical binary pattern-2D and eight wavelet reconstructions |

2D, two-dimensional; 3D, three-dimensional; GLCM, gray-level co-occurence matrix; GLDM, gray-level dependence matrix; GLRLM, gray-level run-length matrix; GLSZM, gray-level size zone matrix; NGTDM, neighbouring gray-tone difference matrix.
